# Insight into the Regulatory Relationships between the Insulin-Like Androgenic Gland Hormone Gene and the Insulin-Like Androgenic Gland Hormone-binding Protein Gene in Giant Freshwater Prawns (*Macrobrachium rosenbergii*)

**DOI:** 10.3390/ijms21124207

**Published:** 2020-06-12

**Authors:** Guang Yang, Zhijie Lu, Zhendong Qin, Lijuan Zhao, Gan Pan, Haiyang Shen, Menglan Zhang, Rishen Liang, Li Lin, Kai Zhang

**Affiliations:** 1 Guangdong Provincial Water Environment and Aquatic Products Security Engineering Technology, Research Center, Guangzhou Key Laboratory of Aquatic Animal Diseases and Waterfowl Breeding, Zhongkai University of Agriculture and Engineering, Guangzhou 510225, China; yangguang@m.scnu.edu.cn (G.Y.); lzjaquaculture@163.com (Z.L.) qinzhendongsc@163.com (Z.Q.); zhaolijuan4234@163.com (L.Z.); 2017022229@m.scnu.edu.cn (H.S.); 2018022517@m.scnu.edu.cn (M.Z.); cheetahliang@126.com (R.L.); 2 Guangdong Provincial Key Laboratory for Healthy and Safe Aquaculture, College of Life Science, South China Normal University, Guangzhou 510631, China; 20091329@m.scnu.edu.cn; 3 Department of Ocean Science, Division of Life Science, The Hong Kong University of Science and Technology, Hong Kong 93117, China

**Keywords:** *Macrobrachium rosenbergii*, insulin-like androgenic gland hormone (IAG), insulin-like androgenic gland hormone-binding protein (IAGBP), RNA interference

## Abstract

Giant freshwater prawns (*Macrobrachium rosenbergii*) are commonly found throughout the world. The size of the male giant freshwater prawn is much larger than that of the female. Therefore, understanding the molecular mechanism that underlies the sexual differentiation of *M. rosenbergii* is of both commercial and scientific importance. Insulin-like androgenic gland hormone (IAG) plays a key role in the differentiation of sex in *M. rosenbergii*. Although IAG has been investigated, the regulatory relationship between IAG and its binding protein partner, the insulin-like androgenic gland hormone-binding protein (IAGBP), has not been studied in *M. rosenbergii*. Here, we cloned and characterized the *IAGBP* from *M. rosenbergii* (*Mr-IAGBP*) for the very first time. Transcriptomic analysis showed that *Mr-IAGBP* mRNA was detected in a wide array of tissues with the highest expression found in the androgenic gland. The importance of *IAG* in male development was further demonstrated by an increase in *IAG* transcripts during the development of the androgenic gland and *Mr-IAG* was only highly transcribed in the androgenic gland of *M. rosenbergii*. Interestingly, we found that the *Mr-IAG* gene expression started during the 20th-day larva after hatching stage (LH20), followed (20th-day post-larval stage, PL20) by a gradual elevation of *Mr-IAGBP* levels. The levels of both genes peaked at the adult stage. The relationship between *Mr*-*IAGBP* and *Mr*-*IAG* was further analyzed using RNA interference. The injection of *Mr-IAGBP* double-stranded RNA (dsRNA) significantly reduced the transcription of *Mr-IAG*, while the amount of *Mr-IAGBP* mRNA and the translation of IAGBP protein was significantly reduced by the injection of *Mr*-*IAG* dsRNA. These results revealed that IAGBP is involved in IAG signaling. Furthermore, our data supports the hypothesis that (IAG and IAGBP)-IAG receptor signaling schemes exist in *M. rosenbergii*. Our results will provide important information for the further study of determining the sex of *M. rosenbergii*.

## 1. Introduction

Giant freshwater prawn (*Macrobrachium rosenbergii*) is a commercially important species of freshwater prawn, which is widely cultured worldwide [1]. Because there has been sexual dimorphism in most economic crustaceans [2], growth in a monosex environment enables them to allocate more energy to growth instead of sexual activity. Thus, there could be a marked rise in the yield and reduction in the length of the reproductive cycle of the *M. rosenbergii* if there is a development of the monosex culture [3].

Males have an exclusive endocrine organ known as the androgenic gland (AG), which was initially observed in crustaceans of swimming blue crab (*Callinectes sapidus*) [4]. Previous studies have proven that AG plays a crucial role in sexual and/or morphotypic differentiation in crustaceans [5,6,7,8,9,10]. Moreover, AG is responsible for masculinity if it is implanted in females and vice versa if removed from males, which exhibits the main role in the differentiation of male sexual characteristics in crustaceans [5,11,12,13,14]. Several androgenic effects of the AG are attributed to another hormone that is encoded by an insulin-like androgenic gland hormone (*IAG*) gene secreted as a proteinaceous hormone [15,16,17,18]. The *IAG* gene expressed specifically in the AG of males in *Cherax quardricarinatus* [15] and *Penaeus monodon* [16]. Recently, an increase in the number of *IAG*s existing in various Decapod species has been recognized [19,20,21]. In addition, a previous study suggested that insulin-like androgenic gland hormone-binding protein (*IAGBP*) modulates and aids the signaling of IAG in the overall route in *Macrobrachium nipponense* [22]. Rosen found that the Insulin-like growth factor-binding protein (IGFBP) in *Cherax quadricarinatus* (Cq-IGFBP) is the first IGFBP family member to specifically interact with IAG [23]. Chandler described in great detail the identification and characterization of the IGFBP in *Sagmariasus verreauxi* (Sv-IGFBP) [24].

The researchers found that silencing of *Mr*-*IAG* led to the cessation of testicular spermatogenesis and spermatogenic cell development in the ampulla of the end of the sperm duct, accompanied by hypertrophy and hyperplasia of the androgenic gland (AG), so it was concluded that *Mr*-*IAG* plays an important role in spermatogenesis and the development of primary and secondary male sexual characteristics in *M. rosenbergii* [18,25,26]. Silencing the *Mr-IAG* gene in *M. rosenbergii* juveniles by the repeated injection of *Mr*-*IAG* double-stranded RNA leads to functional sex reversal [27,28]. In addition, the role of IAG in orange claw male (oc) and blue claw male (bc) morphotype transformation has also been confirmed [29]. Despite the examination of the sequences and expressive profiles of *IAG*, the regulatory mechanism of *IAG* is not fully understood. A previous study suggested that *IAG* and the insulin-like androgenic gland hormone-binding protein (IAGBP) transcription may be regulated by feedback inhibition in crustaceans [22]. Information on the regulatory relationship between *IAG* and *IAGBP* is still limited. Detailed studies on the regulatory relationship between *IAG* and *IAGBP* would help to clarify the signaling schemes of *Mr*-*IAG*. 

The aim of the present study is to investigate the regulatory relationship between *IAG* and *IAGBP* in *M. rosenbergii,* which reflects the roles of these two genes in gonadal development of *M. rosenbergii.* The *IAGBP* gene in *M. rosenbergii* was cloned and characterized using a de novo transcriptomic library. Subsequently, the expression patterns of *Mr*-*IAGBP* and *Mr*-*IAG* in different types of tissues and at different stages of development were investigated. Finally, the relationship between *Mr*-*IAGBP* and *Mr*-*IAG* was demonstrated by gene silencing using double-stranded RNA (dsRNA). Our results clarified the transcriptional relationship between the *Mr*-*IAGBP* and *Mr*-*IAG* genes, which will lay the foundations for further studying of the sexual determination of *M. rosenbergii*.

## 2. Results

### 2.1. Isolation of Full-Length Mr-IAGBP cDNA

**Figure 1:**
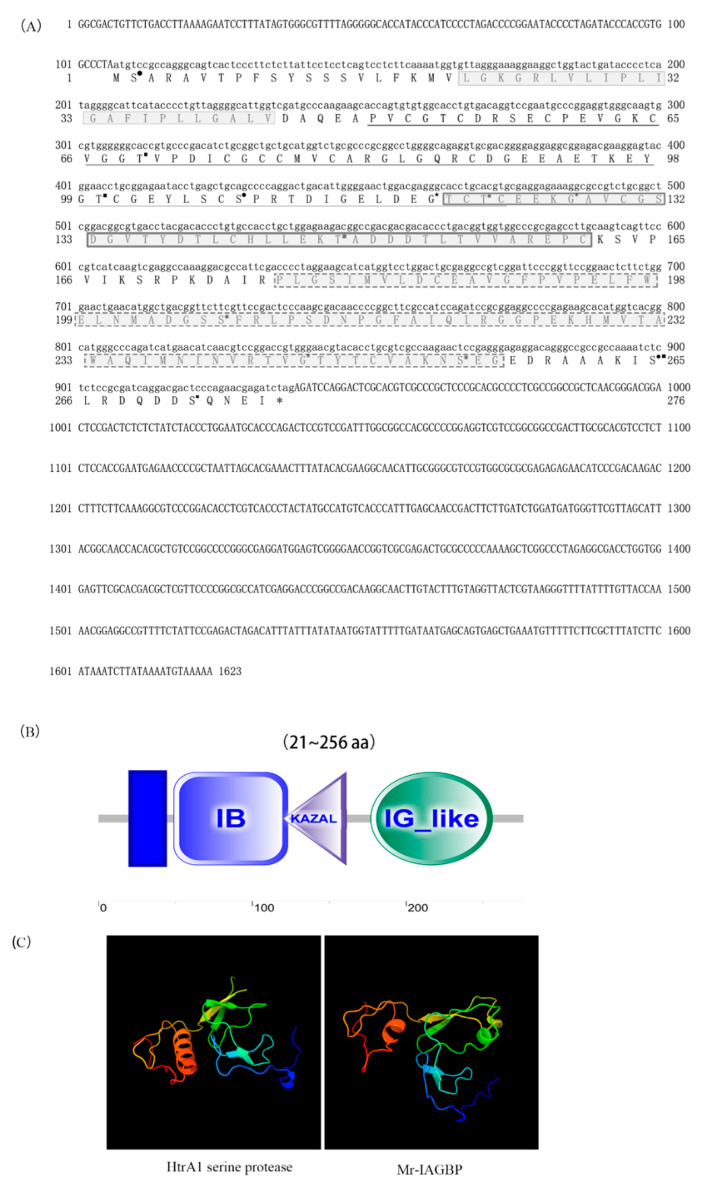
Complete cDNA and protein sequences of *Mr*-*IAGBP*. (**A**) The full-length cDNA sequence comprised 1623 nucleotides (nt), including a 5´-UTR (nt 1–106) and a 3´-UTR (nt 938–1623). The ORF encoded a deduced protein of 276 amino acids. The deduced amino acid sequences are shown with one-letter codes under the coding sequences. The stop codon is indicated by an asterisk. The putative transmembrane region is shown by a box with light colored line and gray background. The IB domain is indicated by a underline, the KAZAL domain is shown by a box with dark colored lines and gray background, and the IG-like domain is indicated by a dotted line with and gray background. The predicted phosphorylation sites, *N*-glycosylation sites, and *O*-glycosylation sites are indicated by black solid squares (■), black solid circles (●), and black solid pentagon (★), respectively. (**B**) Domain architecture organization of Mr-IAGBP (from the N-terminus to the C-terminus) as predicted by SMART (http://smart.embl-heidelberg.de/), showing the transmembrane region and the IB, KAZAL, and IG-like domains. Scale bar represents number of amino acids. (**C**) The comparison of the tertiary structures of Mr- IAGBP and human-IGFBP proteins. The α-helix shows in red, the β-sheet shows in green, and the irregular loop shows in blue.

The full-length *Mr*-*IAGBP* cDNA transcript consisted of 1623 bp (Table S1); where 106 bp belonged to 5’-untranslated region (UTR), and 686 bp to the 3’-UTR, which also included a poly (A) tail. The sequence analysis of the nucleotides exhibited 831 bp from the ORF encodes a 276 aa protein that would weigh 29.42 kDa in molecular weight (Figure 1A). The *Mr*-*IAGBP* gene led to a product that was confirmed to belong to the IGFBP classification. No peptide signal was located. The predicted mature peptide contained seven phosphorylation sites (Thr69, Thr100, Thr122, Thr147, Ser254, Ser265, and Ser272) (Figure 1A). Mature Mr-IAGBP peptide displayed two *O*-glycosylation sites (Gly119, Gly127) and four *N*-glycosylation sites (asparagine, Ser2, Ser108, Ser207, Ser265) (Figure 1A)

**Figure 2:**
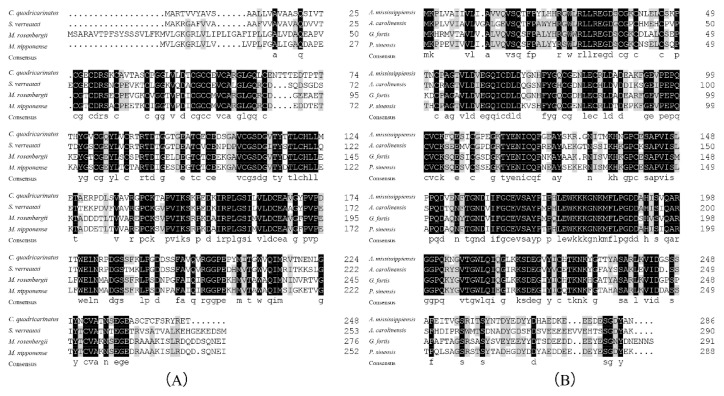
Multiple sequence alignments of Mr-IAGBP amino acid sequences with other crustaceans (**A**), and of IGFBP amino acid sequences between vertebrates (**B**) using ClustalW. Species names are abbreviated at the left and represent: *Macrobrachium nipponense* (KJ831645), *Cherax quadricarinatus* (AGS78412.1), *Sagmariasus verreauxi* (ALZ50690.1), *Alligator mississippiensis* (XP_006263744.1), *Anolis carolinensis* (XP_003215329.1), *Geospiza fortis* (XP_001231917.1), *Pelodiscus sinensis* (XP_006115855.1). The conserved, identical residues are highlighted by black and gray backgrounds.

The encoded transcript was run through the SMART bioinformatics tool, and it revealed that this protein was made up of a trans-membrane region domain (residues 21–43), insulin-like-binding (IB) domain (residues 49–123), a kazal-type serine protease inhibitor (KAZAL) domain (residues 120–161), and an immunoglobulin-like (IG-like) domain (residues 177–256), in this exact order (Figure 1B). The SWISS-MODEL results showed that the three-dimensional structure of IAGBP in *M. rosenbergii* was composed of α-helix and connected random coils, and the protein structure was butterfly-shaped (Figure 1C).The N-terminal conserved motif Cys-Gly-Cys-Cys-Xxx-Xxx-Cys (CGCCXXC) was found to be crucial for insulin-like binding by IGFBP in vertebrates (Figure 2B). A similar determinant (Cys74-Gly75-Cys76-Cys77-Xxx-Xxx-Cys80, CGCCXXC) was identified in the *N*-terminal of Mr-IAGBP (Figure 2A).

### 2.2. Multiple Alignment and Phylogenetic Analysis

**Figure 3:**
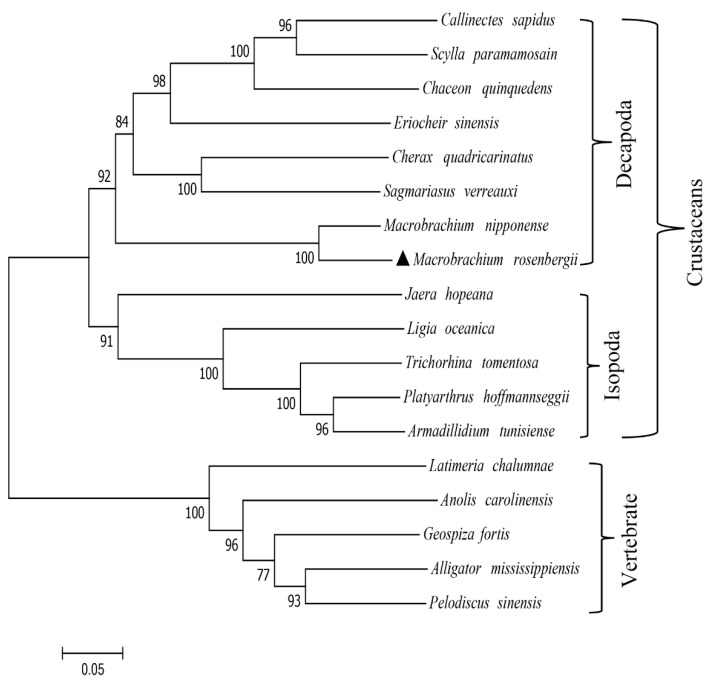
Unrooted phylogenetic tree of Mr-IAGBP. The phylogenetic tree was inferred using the neighbor-joining method with whole, deduced amino acid sequences. The values at the tree nodes are neighbor-joining bootstrap values. The amino acid sequences used for phylogenetic analysis are as follows: *Callinectes sapidus* (AOC31985.1), *Scylla paramamosain* (ALO50698.1), *Chaceon quinquedens* (ART33388.1), *Eriocheir sinensis* (AOE46695.1), *Cherax quadricarinatus* (AGS78412.1), *Sagmariasus verreauxi* (ALZ50690.1), *Macrobrachium nipponense* (AJQ31850.1) *Jaera hopeana* (AYU97982.1), *Ligia oceanica* (AYU97983.1), *Trichorhina tomentosa* (AYU97985.1), *Platyarthrus hoffmannseggii* (AYU97970.1), *Armadillidium tunisiense* (AYU97962.1), *Latimeria chalumnae* (XP_006002507.1), *Anolis carolinensis* (XP_003215329.1), *Geospiza fortis* (XP_005428680.1), *Alligator mississippiensis* (XP_006263744.1), *Pelodiscus sinensis* (XP_006115855.1).

The Mr-IAGBP peptide sequence that was discovered was re-examined in more depth using ClustalW to check for similarities with other crustaceans (Figure 2A). Vertebral IGFBP sequences were also aligned (Figure 2B). The most identical Mr-IAGBP was found with *M. nipponense* (81.16%), and the least identical with *C. quadricarinatus* (48.57%). IGFBP sequences were found to be similar to vertebrates, (at least 65.03%) between the *Anolis carolinensis* and *Geospiza fortis*, and the highest similarity was between *Alligator mississippiensis* and *Pelodiscus sinensis* (79.91%). The results of sequence similarities were constructed with the neighbor-joining method and a phylogenetic tree. Figure 3 demonstrates how two clades of division occur from peptides of the vertebrates and invertebrates: one consists of IAGBP from crustaceans, while the other consists of known IGFBPs from vertebrates and arachnids.

### 2.3. Recombinant Mr-IAGBP Protein Expression, Purification, and Polyclonal Antibodies Analysis

**Figure 4:**
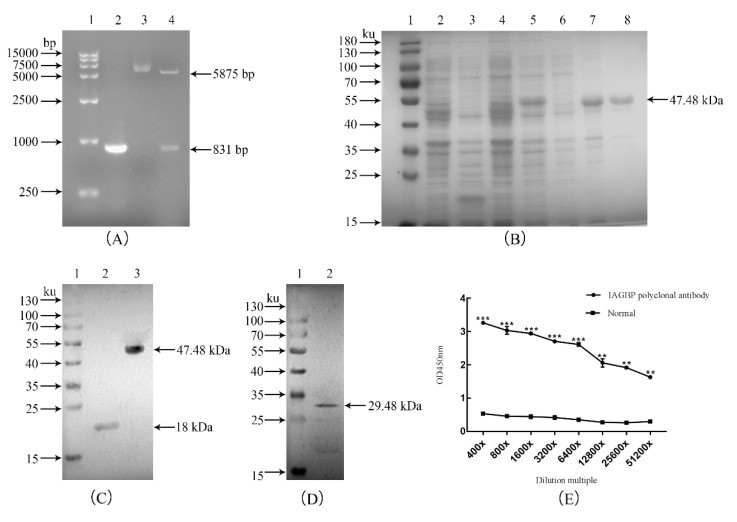
Molecular cloning and expression of *Mr*-*IAGBP* and preparation of polyclonal antibody. (**A**) Cloning of *Mr*-*IAGBP* gene and digestion test of fusion expression vector with restriction enzyme 1. DNA Marker, 2. *Mr*-*IAGBP* PCR fragment, 3. fusion expression vector, 4. enzyme digestion verification. (**B**) Purified recombinant *Mr*-*IAGBP* protein by SDS-PAGE. 1. protein marker, 2. *E.coli* BL21 with pET-32A vector without IPTG, 3. *E. coli* BL21 with pET-32A vector with IPTG, 4. *E.coli* with pET-32A- Mr-IAGBP protein without IPTG, 5. *E.coli* with pET-32A-Mr-IAGBP with IPTG, 6. Supernatant of *E.coli* with pET-32A- Mr-IAGBP, 7. Pellet of *E.coli* with pET-32A-Mr-IAGBP, 8. purified recombinant Mr-IAGBP protein. (**C**) Verification of recombinant Mr-IAGBP protein using His-tag antibody. 1. protein maker. 2. *E.coli* BL21 with pET-32A vector. 3. *E.coli* with pET-32A-Mr-IAGBP. (**D**) Polyclonal antibody of Mr-IAGBP 1. protein marker, 2. total protein of AG tissue. (**E**) Effect detection of the Mr-IAGBP polyclonal antibody (Arabic numerals in the horizontal coordinates represent dilution, ∗∗ represents very significant difference, *p* < 0.01; ∗∗∗ represents extremely significant difference, *p* < 0.001).

The open reading frame (ORF) of *Mr*-*IAGBP* was cloned into a pET-32a vector, transformed to BL21 (DE3), and the recombinant protein fused with His-tag columns was purified and analyzed with SDS-PAGE gel. After double digestion of the recombinant plasmid, two distinct bands appeared, and the size of the target band was consistent with that predicted (Figure 4A). As shown in the SDS-PAGE gel, a distinct band was identified with a size of 47.48 kDa of inclusion bodies (Figure 4B), corresponding to the His-IAGBP recombinant fusion protein (the Mr-IAGBP protein about 29.48 kDa, and the control pET-32a about 18 kDa). Using the His-tag primary antibody, the recombinant Mr-IAGBP protein has a single and distinct band around 47.48 kDa, and the size is as predicted, indicating that the recombinant Mr-IAGBP protein contains a His-tag (Figure 4C). The purified His-IAGBP recombinant protein was injected into the rabbit to produce polyclonal antibodies, which was used as the primary antibody to anti the total protein of the *M. rosenbergii* AG for Western blot. Mr-IAGBP caused the rabbit polyclonal antibodies to react. This, as well as a specific positive band on Western blot nitrocellulose membrane of about 29.48 kDa, ensured the expected molecular weight of Mr-IAGBP protein. This, in turn, also confirmed the successful preparation of the rabbit polyclonal antibodies (Figure 4D). The titer of the polyclonal antibody serum was determined by enzyme-linked immunosorbent assay, and the polyclonal antibody titer was not less than 1:51,200 (Figure 4E).

### 2.4. Tissue and Spatial Distributions of Mr-IAGBP and Mr-IAG Transcripts

**Figure 5:**
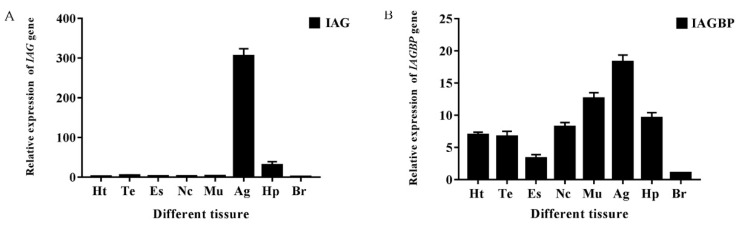
Transcriptional levels of *Mr-IAG* (**A**) and *Mr-IAGBP* (**B**) revealed by qRT-PCR in different tissues. The tissues included: heart (Ht), testis (Te), eyestalk (Es), nerve cord (Nc), muscle (Mu), androgenic gland (Ag), hepatopancreas (Hp), and brain (Br). *Mr-IAG* and *Mr-IAGBP* mRNA levels were normalized to β-actin.

The distributions of tissue of *Mr*-*IAGBP* and *Mr*-*IAG* (Figure 5) were examined by the qRT-PCR method. The *Mr*-*IAGBP* mRNA was found in eight different tissues. The level of transcription was highest in the AG, but similar levels were identified in muscle tissue and the hepatopancreas. Moreover, this value was the lower in the eyestalk (Figure 5B). *Mr*-*IAG* mRNA was located in the AG and hepatopancreas only, where the AG had comparatively higher levels than the hepatopancreas (Figure 5A). In other examined tissues, such as the heart, testis, nerve cord, eyestalk, brain tissue, and muscles, *Mr*-*IAG* expression was not found at all.

**Figure 6:**
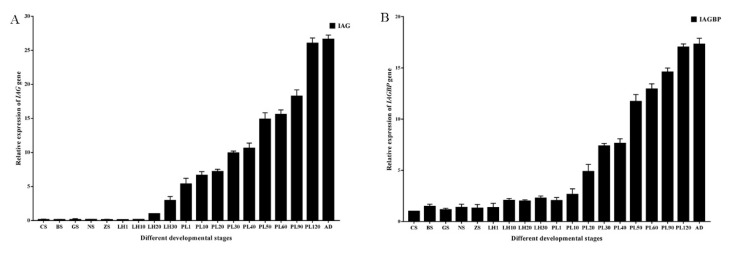
Transcriptional levels of *Mr-IAG* (**A**) and *Mr-IAGBP* (**B**) at different developmental stages, revealed by qRT-PCR. *Mr-IAG* and *Mr-IAGBP* mRNA levels were normalized to β-actin. qRT-PCR data are shown as means ± SE (standard error). CS, cleavage stage; BS, blastula stage; GS, gastrula stage; NS, nauplius stage; ZS, zoea stage; LH1, first-day larva after hatching; LH10, fifth-day larva after hatching; PL1, first-day post-larval stage; PL10, 10th-day post-larval stage; PL20, 20th-day post-larval stage; PL30, 30th-day post-larval stage; PL50, 50th-day post-larval stage; AD, adult stage.

Levels of mRNA in *Mr*-*IAG* and *Mr*-*IAGBP* were calculated while the developmental stage was ongoing from embryo to adult, and this was accomplished using qRT-PCR (Figure 6). We did not detect *Mr*-*IAG* mRNA in embryonic tissues or within 20 days of larva hatching (LH20) stage (Figure 6A), and the expression of *Mr*-*IAG* was detected in post-larval stage and adulthood. Specifically, there was transcription of *Mr*-*IAG* detected at the 20th-day larva after hatching (LH20) stage, and the expression level of *Mr*-*IAG* mRNA from LH20 to the adult stage was always increased. However, the transcription of *Mr*-*IAGBP* were detected from the cleavage stage to adulthood rather than from LH20 to adulthood, and the expression level of *Mr*-*IAGBP* mRNA from PL20 to the adult stage was always increased (Figure 6B).

### 2.5. Transcription in Mr-IAG-dsRNA- and Mr-IAGBP-dsRNA-injection Groups

**Figure 7:**
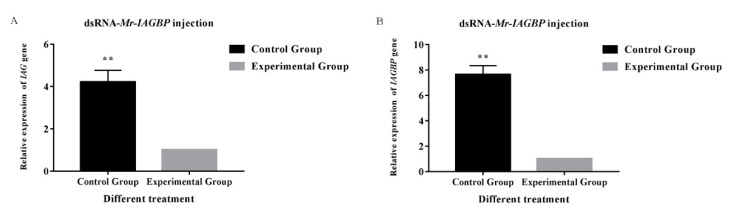
Effects of dsRNA-*Mr*-*IAGBP* injection. (**A**) Effect of *Mr*-*IAGBP* dsRNA on *Mr*-*IAG* mRNA levels in the androgenic gland. (**B**) Effect of *Mr*-*IAGBP* dsRNA on *Mr*-*IAGBP* mRNA levels in the androgenic gland. mRNA levels were analyzed by qRT-PCR. *Mr*-*IAGBP* and *Mr*-*IAG* mRNA levels were normalized to β-actin. qRT-PCR data are shown as means ± SE (standard error). ∗∗*p* < 0.01.

**Figure 8:**
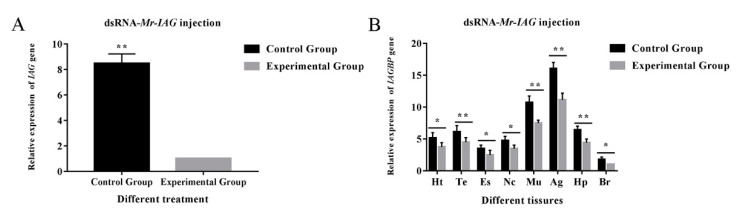
Effects of dsRNA-*Mr*-*IAG* injection. (**A**) Effect of *Mr*-*IAG* dsRNA on *Mr*-*IAG* mRNA levels in the androgenic gland. (**B**) Effects of *Mr*-*IAG* dsRNA on *Mr*-*IAGBP* mRNA levels revealed by qRT-PCR in different tissues. *Mr*-*IAG* and *Mr*-*IAGBP* mRNA levels were normalized to β-actin. The tissues included: heart (Ht), testis (Te), eyestalk (Es), nerve cord (Nc), muscle (Mu), androgenic gland (Ag), hepatopancreas (Hp), and brain (Br). qRT-PCR data are shown as means ± SE (standard error). ∗*p* < 0.05; ∗∗*p* < 0.01.

RNAi was used to describe the correlation between the *Mr*-*IAG* and *Mr*-*IAGBP* in vivo transcriptions. dsRNA was injected into *M. rosenbergii*, along with the target gene or DEPC water. This injection significantly decreased the transcription levels in *Mr-IAG* and *Mr-IAGBP*, as proved by the qRT-PCR (Figure 7 and Figure 8). The mRNA levels in *Mr-IAG* and *Mr-IAGBP* also reduced greatly in the *Mr-IAGBP*-dsRNA-injected group, to 30.5% (Figure 7A) and 15.6% (Figure 7B) of control group prawns, with (*p* < 0.01). *Mr-IAG* mRNA levels were also seen to drop significantly (*p* < 0.01) in the *Mr-IAG*-dsRNA-injected prawns, with a new value of 13.4% (Figure 8A), whereas the *Mr-IAGBP* mRNA levels reduced to 65.6%, 69.7%, and 68.5%, in muscle, the AG, and the epatopancreas, respectively (*p* < 0.01) (Figure 8B). In other tissues (heart, testis, eyestalk, nerve cord, and brain), the mRNA levels were not much different for the two test groups and the control group (Figure 8B).

### 2.6. Western Blotting and Immunohistochemistry Analyses

**Figure 9:**
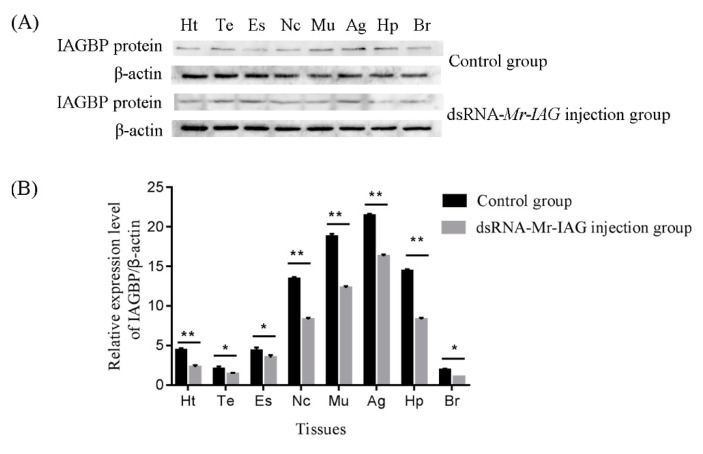
(**A**) The protein of Mr-IAGBP in the different tissues of the control groups and experimental groups by Western blotting. (**B**) Gray value analysis of Mr-IAGBP protein and β-actin protein WB bands in *M. rosenbergii* in the different tissues. The tissues included: heart (Ht), testis (Te), eyestalk (Es), nerve cord (Nc), muscle (Mu), androgenic gland (Ag), hepatopancreas (Hp), and brain (Br).

**Figure 10:**
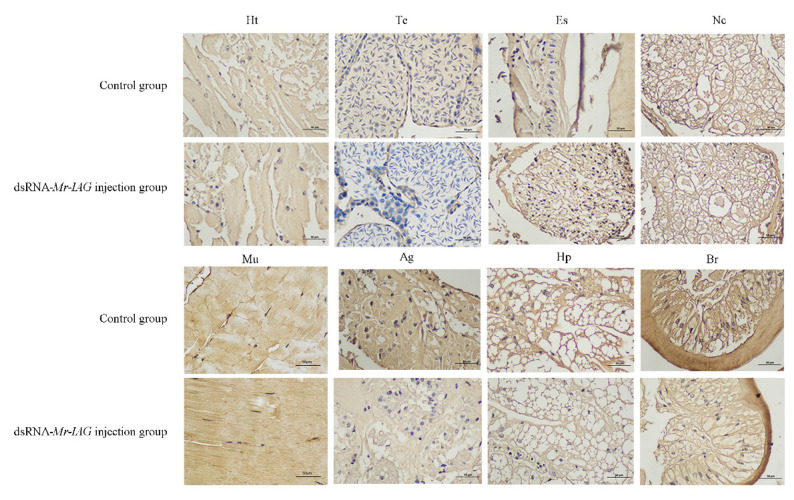
Immunohistochemistry assay of the control groups and experimental groups. The protein of Mr-IAGBP in the different tissues of the control groups and *Mr*-*IAG* dsRNA groups by IHC. The tissues included: heart (Ht), testis (Te), eyestalk (Es), nerve cord (Nc), muscle (Mu), androgenic gland (Ag), hepatopancreas (Hp), and brain (Br).

To further examine the relationship between Mr-IAGBP protein and Mr-IAG protein in *M. rosenbergii*, we examined the expression levels of Mr-IAGBP protein in the heart, testis, nerve cord, eyestalk, androgenic gland, muscle, hepatopancreas, and brain tissues of the control groups (DEPC water) and experimental groups (injection of dsRNA-*Mr*-*IAG*) by Western blotting and immunohistochemistry. Heart, eyestalk, testis, nerve cord, androgenic gland, muscle, hepatopancreas, and brain tissue proteins were extracted from the control groups and experimental groups. Western blotting showed that there were more obvious positive signals in the androgenic gland, hepatopancreas, and muscle tissues of the control groups than in the experimental groups (Figure 9). In addition, the same tissue samples were detected by immunohistochemistry. The results showed that different degrees of positive signals were detected in the heart, testis, eye stalk, nerve cord, muscle, androgen gland, hepatopancreas, and brain tissues in the control groups and experimental groups. The results of immunohistochemistry were consistent with those of Western blotting (Figure 10).

## 3. Discussion

Previously, a great deal of research has focused on the identification and functional expression of *IAG* in *M. rosenbergii* [18,26,30,31]. However, there are still very few studies on the regulatory mechanism of *IAG*. As its binding protein companion, IAGBP may assist the signaling of IAG in the overall route in crustaceans [22]. Therefore, the regulation strategies of *Mr-IAG* and *Mr-IAGBP* at transcriptional and protein levels require further study.

In this study, an *IAGBP* encoding gene was isolated from *M. rosenbergii.* The predicted ORF of *Mr*-*IAGBP* (276 aa) was found to be similar to that of *M. nipponense IAGBP* (GenBank: KJ831645, 252 aa, 81.16% identity). The family of IGFBP precursors are proteins that are rich in cysteine–prepeptides containing 16–20 csyteines–and have the same organization of structure as two conserved domains (N- and C-terminal domains), while the central region, being a variable, separates them [32,33]. The current research identifies the 18 cysteines in the Mr-IAGBP precursor, where the N-terminal domain 12 cysteines are also included, along with four from the variable central region and the C-terminal domain 2. The existence of 12 out of 18 cysteines from the N-terminal domain suggests an increased structure of that domain, which includes a maximum of six disulfide bonds [33]. Mr-IAGBP is seen be surrounded by other crustaceans in the phylogenetic analysis, but the vertebrates created a detached clade. *C. quadricarinatus* [23] and *M. nipponense* [22] gave results that were similar. However, IGFBP sequences were seen to have greater similarity among the vertebrates than among the crustaceans. These differences could be attributed to the various functions of crustaceans as compared to vertebrates.

The expression patterns of *Mr*-*IAGBP* in various tissues suggest that *Mr*-*IAGBP* exhibits the highest expression in the androgenic gland, indicating that this gene is associated with the development of the androgenic gland. In addition, we also discovered a wide distribution of *Mr*-*IAGBP*, which suggests that *IAG* and *IAGBP* may be synthetized in different cell types within a gland and *Mr-IAGBP* may have extensive biological functions. *Mr*-*IAG* was specifically highly expressed in the androgenic gland which is consistent with previous findings [26]. The expression pattern of *Mr*-*IAGBP* and *Mr*-*IAG* at various stages of the development of *M. rosenbergii* was also investigated. Our results showed that *Mr*-*IAG* mRNA was undetected until the LH20 stage during embryonic development. This is different from previous results where *Mr-IAG* was expressed from the PL20 stage [26]. The difference between the two results could be due to the expression levels of *Mr*-*IAG* being detected at more developmental stages. In addition, there was a difference in the results for *M.nipponense* and *Fenneropenaeus chinensis*. *IAG* transcripts were detectable during embryonic development in *M. nipponense* and *F. chinensis* [13,22]. Interestingly, we discovered that levels of *Mr-IAGBP* transcripts increased significantly later (PL20) than those of *Mr-IAG* (LH20). In previous studies, it has been observed that fully functional sex reversal of *M. rosenbergii* males could be achieved by ablation of the androgenic gland into neofemales, and the success rate is greater if the ablation is performed earlier, during PL20 [5,26].

The injection of dsRNA, which targets the *Mr*-*IAGBP* or *Mr*-*IAG*, reduced the transcriptional level of *Mr*-*IAGBP* or *Mr*-*IAG* in correspondence with the degradation of the RNA target. Remarkably, the levels of *Mr*-*IAGBP* mRNA and IAGBP protein were considerably reduced by the injection of *Mr*-*IAG* dsRNA, and *Mr*-*IAG* mRNA levels in the androgenic gland were significantly reduced by the injection of *Mr*-*IAGBP* dsRNA compared to the controls. These results revealed that *IAGBP* is associated with IAG signaling. Previous RNAi studies of *M. rosenbergii* and *C. quadricarinatus* have indicated that *IAG* might regulate its own secretion by feedback inhibition [18,34]. It is therefore possible that *Mr*-*IAG* and *Mr*-*IAGBP* transcription may be regulated through feedback inhibition. Sharabi found that the most significant effect of IAG receptor gene silencing were hypertrophy of AG and the increased production of *Mr*-*IAG*, and abnormally rich immature sperm cells can be seen at the end of sperm [35]. Moreover, Qing confirmed the interactions between IAG receptor gene and IAG in male *Fenneropenaeus chinensis* [36]. Based on the above results, we support the idea that there may be (IAG and IAGBP)-IAG receptor signaling schemes in *M. rosenbergii*.

## 4. Materials and Methods

### 4.1. Tissue Sampling, RNA Isolation, and Reverse Transcription

Both post-larvae (2–3 cm) and adult males (25–30 cm, male external characters observed by the naked eye) of *M. rosenbergii* were taken from Jin Yang Aquaculture Co. Ltd., Guangzhou, China. Each sample was transported to the laboratory for breeding and contained in a 500 L tank of aerated freshwater. The quality of the water was guaranteed by running the 500 L volume through a biofilter. The samples collected from Jin Yang Aquaculture Co. Ltd., Guangzhou, China were divided into six groups. Each group of samples collected included 16 various stages of development, including embryos (zoea stage, blastula stage, nauplius stage, gastrula stage, cleavage stage) [37,38,39], larvae (first, 10th, 20th, and 30th days after larva hatching, 1st, 10th, 20th, 30th, 60th, 90th, and the 120th post-larval days) [40,41], as well as male adults. The sex of the 1st and 20th post-larval day prawns are not distinguishable, therefore, a mixed group was taken. The male prawns of the remaining post-larval days were separated and examined under a microscope to check their sex organs. The sample collection and experiments in the study were carried out in strict accordance with the recommendations of the Laboratory Animals (Ministry of Science and Technology of China 2006) and approved by the Animal Ethics Committee of Zhongkai University of Agriculture and Engineering (Animal Ethics no. 1067, March 6, 2019).

The extracted overall RNA was collected at various developmental stages, as well as separate types of tissues (heart, testis, eyestalk, nerve cord, muscle, androgenic gland, hepatopancreas, and brain) from adult prawns. RNAiso Plus Reagent (TaKaRa, Dalian, China) was used in accordance with the original manufacturer’s protocol. The RNA was isolated and RNase-free DNase I (Sangon, Shanghai, China) treatment was administered to remove any possible contamination by genomic DNA. Using a biophotometer (Eppendorf, Hamburg, Germany), the concentration of the total RNA in each sample was noted, after which 1% agarose gel was used per 2 μL for electrophoresis to evaluate all the sample’s integrity [42]. Each RNA had their first-strand cDNA developed to make real-time reverse transcription-quantitative polymerase chain reaction (qRT-PCR) possible; this was done with 1 μL of the total RNA, 4 μL of 5× iScript reaction mix (Bio-Rad, Hercules, CA, USA), and 1 μL iScript reverse transcriptase in a final volume of 20 μL. The incubation period was 5 min at 85 °C. Reverse transcribed cDNA was stored until needed at 20 °C. 

### 4.2. Gene Cloning of Mr-IAGBP and Bioinformatics Analyses

The transcript fragment of *Mr*-*IAGBP* (Table S1) was first obtained through a de novo transcriptomic library, and with this fragment a complete cDNA of *Mr*-*IAGBP* was cloned by PCR. Table S2 lists every primer used in this study. PCR amplification was executed under the following conditions: 5 min for pre-denaturation at 95 °C, then 35 cycles of 10 s at 95 °C, 30 s at 55 °C, and 1 min at 72 °C, followed by post-extension for 10 min at 72 °C, and then kept at 4 ℃. Electrophoresis was conducted on the PCR fragments with 1% agarose gels. The amplified cDNA fragments were then cloned by insertion into a pMD18-T vector, followed by sequencing with M13 primers in forward or reverse. After verification of the final sequences, they were exposed to the cluster analysis in NCBI [43].

Emboss (http://emboss.Bioinformatics/) and SMART (http://smart.embl-heidelberg.de) tools predicted the protein domains and the complete amino acid (aa) sequence of the *Mr*-*IAGBP* ORF. The BLAST program (http://www.ncbi.nlm.nih.gov/blast) analyzed the similarity in the sequences of the pre-determined nucleotide sequences. CBS prediction servers (http://www.cbs.dtu.dk/services) predicted the signal peptides, *O*-linked and *N*-linked glycosylation sites, and phosphorylation sites. The Clustal X 2.0 program created various alignments of Mr-IAGBP, while the DNAMAN software package (Lynnon Biosoft, Quebec, QC, Canada) generated the breakpoint analyses in detail. The phylogenetic tree was constructed on the basis of ORF aa sequences of Mr-IAGBP proteins by the neighbor-joining (NJ) method [44] using the molecular evolution genetics analysis (MEGA 6.0) software [45]. The bootstrap test was conducted on the basis of 10,000 pseudo-replications to judge the dependability of the phylogenetic tree [46].

### 4.3. Construction of Recombinant Mr-IAGBP Plasmid, Expressing and Purification

In order for the recombinant Mr-IAGBP protein to show phenotypical features, the primers expressed in Table S2 were designed to magnify the full-length ORF of *Mr*-*IAGBP*. The PCR products were disinfected and injected into the pMD-19T vector, and subsequently confirmed by sequencing. After, the 1% agarose gel electrophoresis and target products were decontaminated using a TaKaRa Agarose Gel DNA Purification KitVer.2.0 (TaKaRa, Kyoto, Japan). T4 DNA ligase was used to connect the *Mr*-*IAGBP* fragment to the empty pET-32a vector and kept overnight at 4 ℃. The expression plasmid pET-32a-*Mr*-*IAGBP* was transformed to *E.coli* BL21(DE3) (TIANGEN, Beijing, China) and then cultured in Amp^+^ LB at 37 °C at a speed of 200 rpm. Isopropyl-β-D-thiogalactopyranpside (IPTG) was added to create a final concentration of 0.5 mmol/L and then cultured at 37 °C at a speed of 200 rpm for 4 h, and then centrifuged at 4 °C at a speed of 12,000 rpm for 10 min. Sediment cells were resuspended in PBS and purified with the His Band Resin columns (Sangon Biotech, Shanghai, China), as per protocol. The concentrations of recombinant pET-32a-Mr-IAGBP protein were determined according to the method specified for the Bradford Protein Assay Kit (Beyotime, Shanghai, China). The purified recombinant protein was separated in a 12% SDS-PAGE gel electrophoresis.

### 4.4. Rabbit Polyclonal Antibodies Against Recombinant Mr-IAGBP and Western-blot 

The Frdbio Bioscience & Technology company (Wuhan, China) constructed the rabbit polyclonal antibodies against recombinant protein of Mr-IAGBP. The total protein concentrations from the lysates of tissues were prepared. The total protein quantity was 30 μg, which was divided in 12% SDS-PAGE, and then transferred onto a nitrocellulose membrane (Bio-Rad, Hercules, CA, America). TBST was used containing 137 mM NaCl, 20 mM Tris, and 1% Tween-20, at pH 7.6 to block the membranes, as it contained 3% skimmed milk, and was left for 2.5 h at room temperature. Following this, the membranes were put into incubation overnight at 4 ℃ with the rabbit anti-Mr-IAGBP (1/1000 dilution) primary antibody. The membranes were then rinsed for 5 min in three repetitions with TBST and also incubated with HRP-conjugated secondary antibody goat anti-rabbit IgG (1/10,000 dilution) for 55 min. Another three repeats for 5 min washing with TBST were done [42]. This led to the revealing and measurement of the immunoreactive bands by chemiluminescence (ECL Western Blotting Substrate, Solarbio, Beijing, China) and ChemiScope 6000 (CliNX, Shanghai, China), respectively.

### 4.5. qRT-PCR Analysis of Mr-IAGBP and Mr-IAG 

A quantitative real-time PCR (qRT-PCR) assay in Roche LightCycler 480 (Roche, Branchburg, NJ, USA) was used to calculate the levels of transcription of Mr-IAGBP and Mr-IAG, in the different standards set in this study. Table S2 shows all the primers used. AceQ^®^ qPCR SYBR^®^ Green Master Mix (Vazyme, Nanjing, China) was used to conduct qRT-PCR. The final volume was 20 μL, and this was reacted in three batches, with 1 μL cDN, 1 μL primer of each specificity, 10 μL Green Master Mix, and finally 7 μL ddH_2_O. The conditions provided were 95 °C for 3 min, 40 cycles at 95 °C for 15 s, 60 °C for 30 s, and 72 °C for 20 s, and finally 4 °C for 5 min. The ratio of expression in correlation of the target genes was standardized with an internal reference β-actin gene [42,43], and the 2^−ΔΔCT^ method was used to calculate expression levels of *Mr*-*IAGBP* and *Mr*-*IAG* [47].

### 4.6. Double-Stranded RNA Preparation

A MEGAscript T7 Kit (Ambion, Foster, CA, USA) was used to synthesize dsRNA, and the SnapDragon-dsRNA Design (http://www.flyrnai.org/cgi-bin/RNAi_find_primers.pl) was used to design primers that aid RNA interference (RNAi), with cDNA sequences of *Mr*-*IAGBP* and *Mr*-*IAG* as templates. For the in vitro transcription, the templates were made using gene-specific primers and then PCR, along with the T7 polymerase promoter sequence on their 5´ends (Table S2). To produce 20 µL in vitro transcription, 150 ng of template was used, and the mixture of reaction was incubated for 2 h at 37 °C, after which extraction of synthesized RNA was done with phenol/chloroform. After ethanol precipitation, the RNA was suspended again in DEPC water.

### 4.7. In Vivo Mr-IAG and Mr-IAGBP Silencing

RNAi technology was used to examine the correlation between *Mr*-*IAGBP* and *Mr*-*IAG* at the level of transcription. Three groups of healthy adult prawns (four months post hatching) were made with *n* = 20 for each group, and each wet prawn weighed between 60 g and 80 g. One group was injected with *Mr*-*IAG* dsRNA, one with *Mr*-*IAGBP* dsRNA, and the third with DEPC water, the latter of which was the control group. For two weeks, each prawn was injected twice per week with 5 μg per gram of body weight dsRNA in the sinus of their fifth walking leg [18,26,29,34]. Group two, *Mr*-*IAGBP*-dsRNA, and the control group had their AGs collected for extraction of RNA. From group one, *Mr*-*IAG*-dsRNA, as well as the control group, eight types of tissue were sampled. qRT-PCR was used to define the relative level of mRNA in both the test groups.

### 4.8. Immunohistochemistry Assay

An immunohistochemistry (IHC) assay method was modified for this study and then applied [42,43,48]. In short, various tissues (heart, testis, eyestalk, nerve cord, muscle, androgenic gland, hepatopancreas, and brain) were set at 4 °C for 24 h in 4% paraformaldehyde in paraffin. At the end, the samples were divided into sections of 4 μm and then baked for 2 h at 60 °C. These sections were deparaffinized after being shifted to slides by applying xylene, after which they were rehydrated. To retrieve antigens, these slides were all submerged in a buffer of EDTA used for antigenic retrieval and then put into the microwave for 15 min. After this, 3% hydrogen peroxide (H_2_O_2_) in methanol was applied, and the slides were incubated for 1 h with bovine serum albumin (BSA) at room temperature to prevent non-specific bonding. These tissue sections were again incubated in 1% blocking solution (1/200 dilution) with rabbit anti-Mr-IAGBP primary antibody for a whole night at 4 °C. In the morning, three washes with TBST were done, and the glass slides were incubated for 50 min with HRP-conjugated goat anti-rabbit IgG (1/1000 dilution) at standard room temperature. A DAB substrate solution was used to develop them (Guge Biotech, Wuhan, China). The final step was to counterstain the sections with hematoxylin before mounting and photographing them (ECLIPSE E100, Nikon, Tokyo, Japan).

### 4.9. Statistical Analysis

All data were reported in the form of means ± SE (standard error; *n* = 9). SPSS 22.0 software was used to analyze the statistics, and the statistical significance was given by one-way ANOVA, after which Duncan’s multiple range test was done. Significance was set at *p* < 0.05.

## 5. Conclusions

To summarize, we first cloned and characterized the *Mr-IAGBP*. Multiple sequence alignment and phylogenetic analyses revealed the association and evolutionary relationship of the *IAGBP* genes with both the vertebrate and invertebrate species. We further demonstrated that the important roles of *Mr*-*IAGBP* throughout androgenic gland development and the levels of *Mr*-*IAG* transcripts increased earlier (LH20) than those of *Mr-IAGBP* (PH20). Furthermore, RNA interference revealed that there may be a positive regulatory relationship between *Mr*-*IAG* and *Mr*-*IAGBP* in *M. rosenbergii*. Therefore, we suggest that *Mr*-*IAG* in combination with *Mr*-*IAGBP* may help regulating the development of androgenic gland tissue in *M. rosenbergii*. Our data support the hypothesis that (IAG and IAGBP)-IAG receptor signaling schemes exist in *M. rosenbergii*.

## Supplementary Materials

The following are available online at https://www.mdpi.com/1422-0067/21/12/4207/s1, the following are available online: Table S1, The sequence information for full-length *Mr-IAGBP* cDNA. Table S2, primers used in the present study.

## Abbreviations

AG	Androgenic gland
IGFBP	Insulin-like growth factor-binding protein
ORF	Open reading frame
PBS	Phosphate buffer saline
TBST	Tris buffered saline tween
HRP	Horseradish Peroxidase
BSA	Bovine serum albumin
EDTA	Ethylene diamine tetraacetic acid
DEPC	Diethyl pyrocarbonate
