# Supplementary material for: Insight into the Regulatory Relationships between the Insulin-Like Androgenic Gland Hormone Gene and the Insulin-Like Androgenic Gland Hormone-binding Protein Gene in Giant Freshwater Prawns (Macrobrachium rosenbergii)
Source: Int J Mol Sci. 2020 Jun 12;21(12):4207. doi: 10.3390/ijms21124207 (PMC7352508; doi:10.3390/ijms21124207)
Supplement: Supplementary file 1 [file ijms-21-04207-s001.zip › Table S1.docx]

**Table S1** The sequence information for full-length *Mr-IAGBP* cDNA

ATGTCCGCCAGGGCAGTCACTCCCTTCTCTTATTCCTCCTCAGTCCTCTTCAAAATGGTGTTAGGGAAAGGAAGGCTGGTACTGATACCCCTCATAGGGGCATTCATACCCCTGTTAGGGGCATTGGTCGATGCCCAAGAAGCACCAGTGTGTGGCACCTGTGACAGGTCCGAATGCCCGGAGGTGGGCAAGTGCGTGGGGGGCACCGTGCCCGACATCTGCGGCTGCTGCATGGTCTGCGCCCGCGGCCTGGGGCAGAGGTGCGACGGGGAGGAGGCGGAGACGAAGGAGTACGGAACCTGCGGAGAATACCTGAGCTGCAGCCCCAGGACTGACATTGGGGAACTGGACGAGGGCACCTGCACGTGCGAGGAGAAAGGCGCCGTCTGCGGCTCGGACGGCGTGACCTACGACACCCTGTGCCACCTGCTGGAGAAGACGGCCGACGACGACACCCTGACGGTGGTGGCCCGCGAGCCTTGCAAGTCAGTTCCCGTCATCAAGTCGAGGCCAAAGGACGCCATTCGACCCCTAGGAAGCATCATGGTCCTGGACTGCGAGGCCGTCGGATTCCCGGTTCCGGAACTCTTCTGGGAACTGAACATGGCTGACGGTTCTTCGTTCCGACTCCCAAGCGACAACCCCGGCTTCGCCATCCAGATCCGCGGAGGCCCCGAGAAGCACATGGTCACGGCATGGGCCCAGATCATGAACATCAACGTCCGGACCGTGGGAACGTACACCTGCGTCGCCAAGAACTCCGAGGGAGAGGACAGGGCCGCCGCCAAAATCTCTCTCCGCGATCAGGACGACTCCCAGAACGAGATCTAG
