# Supplementary material for: Insight into the Regulatory Relationships between the Insulin-Like Androgenic Gland Hormone Gene and the Insulin-Like Androgenic Gland Hormone-binding Protein Gene in Giant Freshwater Prawns (Macrobrachium rosenbergii)
Source: Int J Mol Sci. 2020 Jun 12;21(12):4207. doi: 10.3390/ijms21124207 (PMC7352508; doi:10.3390/ijms21124207)
Supplement: Supplementary file 1 [file ijms-21-04207-s001.zip › Table S2.docx]

**Table S2.** Primers used in the present study.

| Primers | Sequences (5'-3') | Purpose |
| --- | --- | --- |
| *IAG*-F | CCGAGATCAAGTGTGTGTTGTTC | qRT-PCR |
| *IAG*-R | TATAGATGTCAGCAGATCGTCGC |  |
| *IAGBP*-F | ATGTCCGCCAGGGCAGTCACTC | RT-PCR |
| *IAGBP*-R | CTAGATCTCGTTCTGGGAGTCGTCC |  |
| *IAGBP*-F2 | GGAACTCTTCTGGGAACTGAACA | qRT-PCR |
| *IAGBP*-R2 | CGGACGTTGATGTTCATGATCTG |  |
| *IAGBP*-F3 | CGCGGATCCATGTCCGCCAGGGCAGTCACTC | recombinant plasmid |
| *IAGBP*-R3 | CCCAAGCTTCTAGATCTCGTT CTGGGAGTCGTCC |  |
| β-actin-F | GTCGTGACTTGACCGATTACCT | qRT-PCR |
| β-actin-R | ATCTCCTGCTCGAAGTCCAATG |  |
| ds-IAG-F | GCGTAATACGACTCACTATAGGGGCGACATAACGAACACCCTT | ds-*IAG* primer for RNAi |
| ds-IAG-R | GCGTAATACGACTCACTATAGGGATGCAATATTCGGCGACTTC |  |
| ds-IAGBP-F | GCGTAATACGACTCACTATAGGGGGTGTTAGGGAAAGGAAGGC | ds-*IAGBP* primer for RNAi |
| ds-IAGBP-R | GCGTAATACGACTCACTATAGGGATGCTTCCTAGGGGTCGAAT |  |

Note: T7 polymerase promoter sequence is underlined.
